# rawMSA: End-to-end Deep Learning using raw Multiple Sequence Alignments

**DOI:** 10.1371/journal.pone.0220182

**Published:** 2019-08-15

**Authors:** Claudio Mirabello, Björn Wallner

**Affiliations:** IFM Bioinformatics, Linköping University, Linköping, Sweden; University of Michigan, UNITED STATES

## Abstract

In the last decades, huge efforts have been made in the bioinformatics community to develop machine learning-based methods for the prediction of structural features of proteins in the hope of answering fundamental questions about the way proteins function and their involvement in several illnesses. The recent advent of Deep Learning has renewed the interest in neural networks, with dozens of methods being developed taking advantage of these new architectures. However, most methods are still heavily based pre-processing of the input data, as well as extraction and integration of multiple hand-picked, and manually designed features. Multiple Sequence Alignments (MSA) are the most common source of information in *de novo* prediction methods. Deep Networks that automatically refine the MSA and extract useful features from it would be immensely powerful. In this work, we propose a new paradigm for the prediction of protein structural features called rawMSA. The core idea behind rawMSA is borrowed from the field of natural language processing to map amino acid sequences into an adaptively learned continuous space. This allows the whole MSA to be input into a Deep Network, thus rendering pre-calculated features such as sequence profiles and other features calculated from MSA obsolete. We showcased the rawMSA methodology on three different prediction problems: secondary structure, relative solvent accessibility and inter-residue contact maps. We have rigorously trained and benchmarked rawMSA on a large set of proteins and have determined that it outperforms classical methods based on position-specific scoring matrices (PSSM) when predicting secondary structure and solvent accessibility, while performing on par with methods using more pre-calculated features in the inter-residue contact map prediction category in CASP12 and CASP13. Clearly demonstrating that rawMSA represents a promising development that can pave the way for improved methods using rawMSA instead of sequence profiles to represent evolutionary information in the coming years. **Availability**: datasets, dataset generation code, evaluation code and models are available at: https://bitbucket.org/clami66/rawmsa.

## 1 Introduction

Predicting the 3D-structure of a protein from its amino acid sequence has been one of the main objectives of structural bioinformatics for decades now [1], yet a definite solution has not been found yet. The most reliable approaches currently involve *homology modeling*, which allows a known protein structure to be assigned an unknown protein provided that there is a detectable sequence similarity between the two. When homology modeling is not viable, *de novo* techniques are needed, either based on physical-based potentials [2] or knowledge-based potentials [3–6]. In the first case, an energy function is used to estimate the free energy of a given protein conformation along with a search function that tries different conformations in order to minimize the energy function [7]. Unfortunately, even small relatively small proteins have many degrees of freedom making it prohibitively expensive to fold them even on customized computer hardware [8]. Knowledge-based potentials, on the other hand, can be learned using statistics [3] or machine learning [9] to infer useful information from known examples of protein structures. This information can be used to constrain the problem, thus greatly reducing the conformational search space and enable prediction of larger proteins and complexes.

In the last couple of decades, a variety of machine learning methods have been developed to predict a number of structural properties of proteins: secondary structure (SS) [10–15], relative solvent accessibility (RSA) [15–18], backbone dihedrals [19], disorder [15, 20, 21], disorder-to-order transition [22, 23], contact maps [24–27], and model quality [9, 28–30].

The most important information used by most (if not all) methods above is a multiple sequence alignment (MSA) of sequences homologous to the target protein. The MSA consists of aligned sequences and to allow for comparisons and analysis of MSAs, they are often compressed into position-specific scoring matrices (PSSM), also called sequence profiles, using the fraction of occurrences of different amino acids in the alignment for each position in the sequence. The sequence profile describes the available evolutionary information of the target protein and is better than a single sequence representation, often providing a significant increase in prediction accuracy [31, 32]. An obvious limitation of compressing an MSA into a PSSM is the loss of information that could be useful to obtain better predictions. Another potential issue is that whenever the MSA contains few sequences, the statistics encoded in the PSSM will not be as reliable and the prediction system may not be able to distinguish between a reliable and an unreliable PSSM.

SS, RSA and similar structural properties are sometimes used as intermediate features to constrain and guide the prediction of more complex properties in a number of methods [33–35]. An example of this comes from the methods used for the prediction of inter-residue contact maps, where evolutionary profiles are integrated with predicted SS and RSA to improve performance [36–38].

More recently, contact map prediction methods have been at the center of renewed interest after the development of a number of techniques to analyze MSAs in search of direct evolutionary couplings. These methods have led to a big leap in the state of the art [39–41]. However, their impressive performance is correlated with the number of sequences in the MSA, and is not as reliable when few sequences are related to the target. This means that evolutionary coupling methods have not completely replaced older machine learning-based systems, but have been integrated, usually in the form of extra inputs, along with the previously mentioned sequence profiles, SS and RSA, into even more complex machine learning systems. At the same time, the Deep Learning has proved to be a useful tool for better integrating the growing number and complexity of input features [42–45].

However, one might argue that this kind of integrative approach, combining individually derived features, ignores a key aspect of deep learning, i.e. that features should be automatically extracted by the network rather than being provided to the network as inputs [46]. If we wanted to take full advantage of deep learning by using it in the same way it is employed for tasks such as image classification, one idea could be to provide a raw MSA input. Since the MSA is the most basic, lowest level input that methods use, it would make sense not to compress it into profiles, but instead let the deep network extract features as part of the training. However, a MSA is not an image or an audio track, and there is no native way of feeding such a large block of strings as input to a deep network.

In this work we try to overcome this hurdle and introduce a new system for the *de novo* prediction of structural properties of proteins called rawMSA. The core idea behind rawMSA borrowed from the field of natural language processing a technique called *embedding* [47], which we use to convert each residue character in the MSA into a floating-point vector of variable size. This way of representing residues is adaptively learned by the network based on *context*, i.e. the structural property that we are trying to predict. To showcase the idea, we designed and tested several deep neural networks based on this concept to predict SS, RSA, and Residue-Residue Contact Maps (CMAP).

## 2 Methods

### 2.1 Inputs

Unlike the classical machine learning methods for the prediction of protein features, rawMSA does not compress the Multiple Sequence Alignment into a profile but, rather, uses the raw aligned sequences as input and devolves the task of extracting useful features to the deep network. The input to the deep network is a flat FASTA alignment. Before it is passed to the input layer of the neural network, each letter in the input is mapped to an integer ranging from 1 to 25 (20 standard residues plus the non-standard residues *B*, *U*, *Z*, *X* and − for gaps). If the alignment of a protein of length *L* contains *N* sequences, including the target, or “master” sequence, it is translates to an array of *L* × *N* integers. The master sequence occupies the first row of the array, while the following rows contain all the aligned sequences, in the order of output determined by the alignment software. Since MSAs for large protein families can contain up to tens of thousands of sequences, a threshold is set so that no more than *Y* sequences are used. For details on the alignment depth threshold, see the “Architecture” section.

When training on or predicting SS or RSA, a sliding window of width 31 is applied to the MSA so that *L* separate windows of size 31 × *Y*, one for each residue in the master sequence, are passed to the network. The central column in the window is occupied by the residue in the master sequence for which a prediction is being made and the corresponding aligned residues from the other sequences. Zero-padding is applied at the N- and C- terminals of the master sequence or if the master sequence is shorter than the window size, and at the bottom if the number of aligned sequences is smaller than the maximum alignment depth *Y*. Note that residues are mapped to integers larger than zero and do not interfere with zero-padding.

### 2.2 Architecture

**Fig 1:**
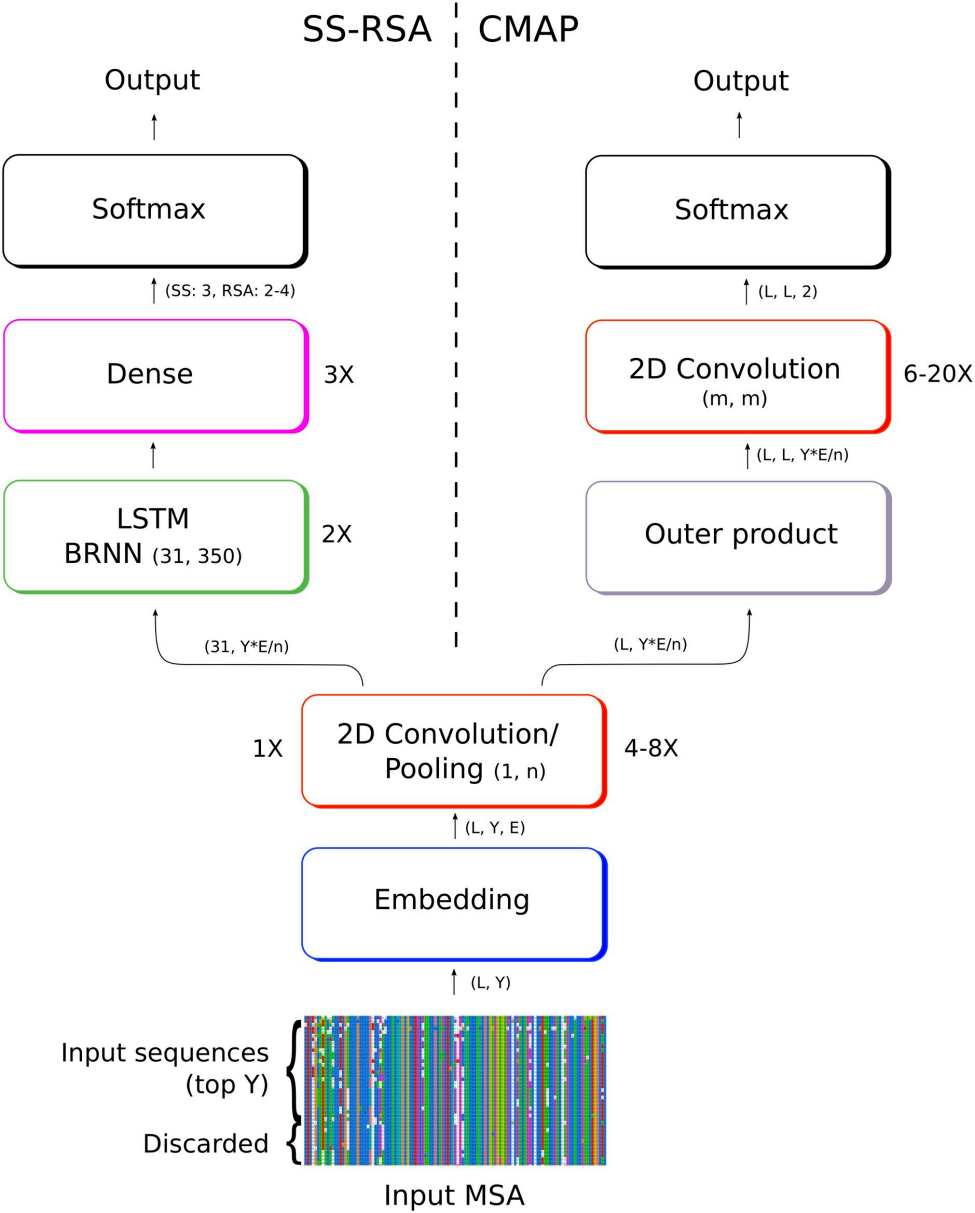
Network architecture for two rawMSA networks. On the left, the *SS-RSA* network predicts the secondary structure and relative solvent accessibility of each amino acid; on the right the *CMAP* network predicts the full contact map of the protein. The first layers are in common between the SS-RSA and CMAP architectures, although with slightly different settings, and provide the basis for the rawMSA approach.

We developed two different architectures for three different applications *SS-RSA* for the SS and RSA prediction and *CMAP* for the contact map prediction. In Fig 1 we show an example of the network architecture. The networks trained in this work might use different numbers of convolutional, fully connected or BRNN layers, as well as slightly different parameters, but they all share this same basic overall structure.

Since with rawMSA we abandoned the use of sequence profiles, which are also useful to represent the amino acid information in a computer-friendly format, i.e. a matrix of floating points, we needed to come up with a way of representing the input. In this case, where the inputs can be very large (up to hundreds of thousands of amino acids), categorical data cannot be translated with sparse, memory inefficient techniques such as *one-hot encoding*.

To resolve this issue, the first layer of rawMSA is a trained, shallow two-layer neural network called an *embedding layer*. Embeddings are a compact way of representing a discrete set of inputs into a continuous space [47]. This technique is widely used in natural language processing where the inputs are made of a sequence of words taken from a dictionary and mapped to an n-dimensional space in a vector of floats of size *n*. When dealing with word embeddings in natural language, words that represent similar concepts, at least in a certain context, will be in close proximity in the output space. A similar idea might also be very useful when dealing with the discrete set of amino acids [48], since they also share context-dependent similarities. For example, when it comes to the context of secondary structure, if we look at the Chou-Fasman amino acid propensities table [49], glutamic acid and methionine are both strongly associated with alpha-helices, so it might be useful that such amino acids are represented by similar vectors when predicting secondary structure.

The embedding layers in rawMSA output a vector of size *E* ranging from from 10 to 30, depending on the model, for each input residue in the alignment. In general, we have found that larger embedding vectors tend to give better results. The embedding layer is used both in the SS-RSA and the CMAP networks.

#### 2.2.1 SS-RSA

In the case of SS-RSA, a 2D convolutional layer is stacked on top of the embedding layer, followed by a max pooling layer. The convolutional layer has a number of filters equal to the dimensionality of the embedding space. The convolution filters have the shape of column vectors, rather than square matrices as is usually the case, thus the size of the convolution windows varies between 1 × 10 to 1 × 30 depending on the model. This means that convolution is performed along each column in the MSA and the information does not spread across columns (i.e. across adjacent residues in the input sequence). Pooling is performed selecting the maximum value in a window of the same size. In this way, if the dimension of the input is 31 residues by 500 alignments before embedding and 31 × 500 × 10 after embedding, this is reduced to a vector of size 31 × 50 × 10 following the convolution and pooling layers, if the convolution and pooling windows are of size 1 × 10. The convolutional and pooling layers are followed by a stack of two Long Short-Term Memory (LSTM) bidirectional recurrent layers, where each LSTM module contains 350 hidden units. The final three layers are fully connected, with softmax layers to output the classification prediction for SS (3 classes: Helix, Strand, Coil) or RSA (4 or 2 classes), depending on the model. Dropout is applied after each recurrent or dense layer to avoid overfitting, with variable fractions of neurons being dropped depending on the model (0.4 to 0.5). All the convolutional layers have ReLU activations and the outputs are zero-padded to match the two first dimensions of the inputs.

#### 2.2.2 CMAP

For CMAP, the network predicts a whole contact map of size *L* × *L* for a protein of length *L*. The input, in this case, is not split in windows, but we use the whole width MSA at once, while the depth is cut at the *Y* top alignments. The network for the first part of CMAP is similar to SS-RSA, with an embedding layer followed by up to six (rather than only one) 2D convolution/max pooling layers along each MSA column. In this case though, the output of the network is a contact map with shape *L* × *L*, so the preceding layers should represent the interaction between pairs of residues. This change of dimensionality is performed with a custom layer that performs the outer product from the output of the first stack of convolutional layers (*H*):

$$\begin{array}{c} OP=\hat{H}\bar{H} \end{array}$$ (1)

Where *H* has dimensionality (*L* × *F* × *S*), $\hat{H}$ and $\bar{H}$ are obtained by adding singleton dimensions to *H* ((*L* × 1 × *F* × *S*) and (1 × *L* × *F* × *S*) respectively).

This operation generates a four-dimensional hidden tensor *OP* of shape *L* × *L* × *F* × *S*, where *F* and *S* are the last two dimensions of the hidden tensor before the outer product. This output is then reshaped to a three-dimensional tensor of shape *L* × *L* × (*F* * *S*) and passed to a new stack of six to 20 (depending on the model) 2D convolutional layers with squared convolutions of varied size (3x3, 5x5, 10x10) and number of filters (10 to 50). The last convolutional layer has shape *L* × *L* × 2 and is followed by a softmax activation layer that output the contact prediction with a probability from 0 to 1. All the convolutional layers have ReLU activations and the outputs are zero-padded to match the two first dimensions of the inputs. Batch normalization is performed in the outputs of the convolutional layers in the CMAP network.

### 2.3 Training

rawMSA was implemented in Python using the Keras library [50] with TensorFlow backend [51]. Training and testing were performed on computers equipped with NVIDIA GeForce 1080Ti, Tesla K80, and Quadro P6000 GPUs.

The training procedure was run including one protein in each batch block, regardless of the size, and using an RMSprop optimizer with sparse categorical cross-entropy as loss function. The SS-RSA network was trained for five epochs, while the CMAP network was trained for up to 200 epochs. During training, a random 10% of the training samples were reserved for validation, while the rest were used for training. After training, the model with the highest accuracy on the validation data was used for testing.

### 2.4 Data sets

The data set is composed of protein chains extracted from a 70% redundancy-reduced version of PDB compiled by PISCES [52] in April 2017 with minimum resolution of 3.0 Å and R-factor 1.0. This set contains 29,653 protein chains.

#### 2.4.1 Avoiding homolog contamination

When training our networks, we want to make sure that the testing and training sets are rigorously separated so that no protein in the test set is too similar to any protein that the network has already “seen” during the training phase.

While most secondary structure and solvent accessibility prediction methods have been using 25-30% sequence identity as the threshold to separate testing and training sets [53–56], this practice has been discouraged as it has been shown that it is not sufficient to avoid information leakage [57]. This is apparently also valid for raw MSA inputs and ur tests separating sets at 25% sequence identity yields higher accuracies compared to our final results (data not shown).

To correctly split training and testing sets, we used two databases based on a structural classification of the proteins: ECOD [58] and SCOPe [59] databases to assign one or more superfamilies to each of the protein chains in the initial set. Then, we removed any chains that were related to more than one superfamily. The set generated from ECOD contains 16,675 proteins (ECOD set), while the one generated from SCOPe contains 9885 proteins (SCOPe set). The SCOPe set contains fewer proteins than the ECOD set since SCOPe has a lower coverage of the PDB. We split each set into five subsets by making sure that no two proteins from the same superfamily were placed in two separate subsets. This ensured that the respective MSA inputs would not be too similar to each other and is the recommended practice when training neural networks using sequence profiles, which are extracted from MSAs [57].

We used the SCOPe subsets to perform five-fold cross-validation on SS-RSA. We also used one of the ECOD subsets to train and validate CMAP, where the validation set was used to determine when to stop training to avoid overfitting, and to select the models that would be ensembled and tested, i.e. the models with the lowest validation error.

#### 2.4.2 Multiple Sequence Alignments

The MSAs for both SS-RSA and CMAP were obtained with HHblits [60] by searching with the master sequence against the HMM database clustered at 20% sequence identity from February, 2016 for three iterations, with 50% minimum coverage, 99% sequence similarity threshold, and 0.001 maximum E-value. We also obtained a second set of MSAs by running JackHMMER [61], for three iterations and 1*e* − 3 maximum E-value on the UniRef100 database from February 2016. The HHblits alignments were used to train and test the SS-RSA networks. The HHblits alignments were also used to train the CMAP network, while both the HHblits and the JackHMMER alignments were used as inputs when ensembling CMAP networks (see Ensembling section), as it improved the prediction accuracy. This approach was also tried for the SS-RSA network, but no improvement was observed.

#### 2.4.3 Test sets

We labeled the CMAP data sets by assigning a native contact map to each protein. A contact was assigned to a pair of residues in a protein if the Euclidean distance between their *Cβ* atoms (*Cα* for Gly) in the crystal structure from the PDB was lower than 8 Å. Otherwise, the two residues were assigned the non-contact label.

We tested CMAP on the CASP12 RR (Residue-Residue) benchmark [62], which is composed of 37 protein chains/domains of the Free Modeling class (FM), i.e. protein targets for which no obvious protein homologs could be found at the time of the experiment (May-August 2016). To ensure a fair comparison with the predictors which participated in CASP12, we performed the benchmark in the same conditions to which all the other predictors where subjected at the time of the CASP experiment. We made sure that all protein structures (from Apr, 2016) in the training set (Apr, 2016) and sequences (from Feb, 2016) in the HHblist HMM and UniRef100 databases were released before CASP12 started.

To test SS-RSA, we calculated the secondary structure (SS) and the Relative accessible Surface Area (RSA) with DSSP 2.0.4 [63]. We reduced the eight SS classes (G, H, I, E, B, S, T, C) to the more common three classes: Coil, Helix, Extended (C, H, E). We used the theoretical Maximum Accessibility Surface Area (Max ASA) defined in [64] to calculate the RSA from the absolute surface areas (ASA) in the DSSP output and we used [0, 0.04], (0.04, 0.25], (0.25, 0.5], (0.5, 1] as thresholds for the four-class RSA predictions (Buried, Partially Buried, Partially Accessible, Accessible), and [0, 0.25], (0.25, 1] as thresholds for the two-class RSA predictions (Buried, Accessible). We discarded the proteins for which DSSP could not produce an output, as well as those that had irregularities in their PDB formats. The final set contained 9,680 protein chains.

#### 2.4.4 Quality measures

The measure of the performance of the trained ensemble of SS-RSA networks is the three-class accuracy (Q3) for SS and the four-class and two-class accuracy for RSA, which are calculated by dividing the number of correctly classified residues by the total number of residues in the dataset.

CMAP predictions for the CASP12 RR benchmark set were evaluated in accordance with the CASP criteria by calculating the accuracy of the top *L*/5 predicted long-range contacts, where *L* is the length of the protein, and the long-range contacts are contacts between residues with sequence separation distance over 23.

#### 2.4.5 Ensembling

Ensembling models usually yield a consensus model that performs better than any of the networks included in the ensemble [65]. Several networks both for CMAP and SS-RSA have been trained with different parameters (see “Results” section). Even though some models have worse performances on average, they are still saved. All the saved models that have been trained on the same set are used at testing time. The outputs from each model are ensembled to determine the final output. This is done by averaging all outputs from the softmax units and selecting the final class by picking the class with the highest average probability.

In the CMAP case, each model in the ensemble is used to make two predictions for each target using either HHblits or JackHMMER alignment. Although the CMAP network is trained only on HHblits alignments, using the JackHMMER alignments in the ensemble improved the overall accuracy of the predictor.

## 3 Results and discussion

### 3.1 Embeddings

**Fig 2:**
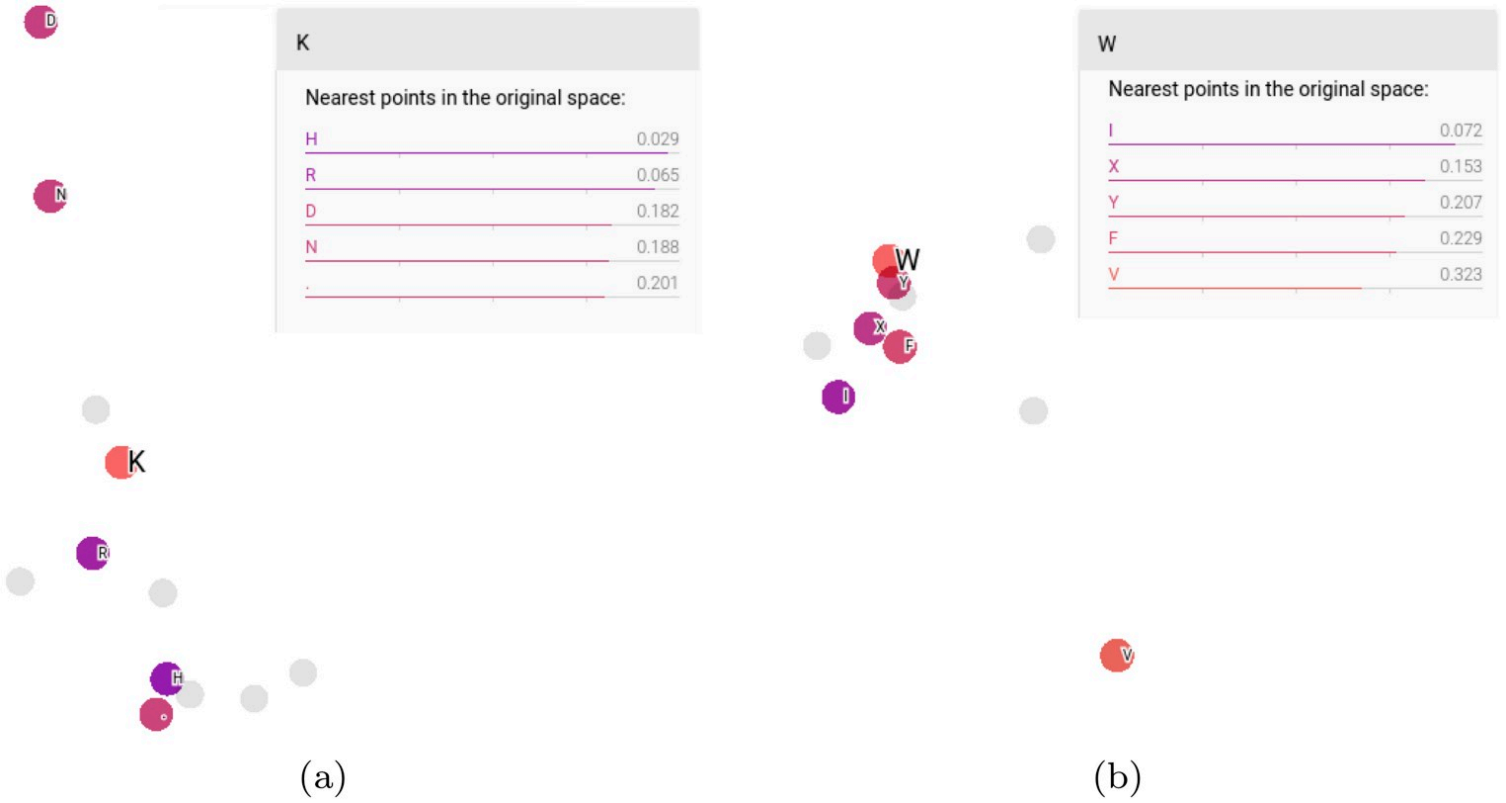
2D PCA of the space of the embedded vectors representing the single residues. In this example, we show the embedding outputs of a simpler network where the original space has a dimensionality of four. The residues that are closest (lower cosine between the 4D vectors) to (a) lysine and (b) tryptophan are colored (the closer the residue, the darker the hue).

Tensorboard in Tensorflow can be used to visualize the output of the embeddings layer to see how each residue type is mapped on the output space. In Fig 2 we show the embeddings outputs for an early version of the SS-RSA network where each amino acid type is mapped on a 4D space. The 4D vectors are projected onto a 2D space by principal component analysis on Tensorboard. In the figure, the amino acids that are the closest (lowest cosine between the 4D vectors) to lysine (Fig 2a) and tryptophan (Fig 2b) are highlighted. The amino acids closest to lysine (K) are histidine (H) and arginine (R), which makes sense, since they can all be positively charged. Similarly, the residues closest to the hydrophobic tryptophan (W) are also hydrophobic, indicating that the embeddings can discriminate between different kinds of amino acids and map them onto a space that makes sense from a chemical point of view.

### 3.2 SS and RSA predictions

**Fig 3:**
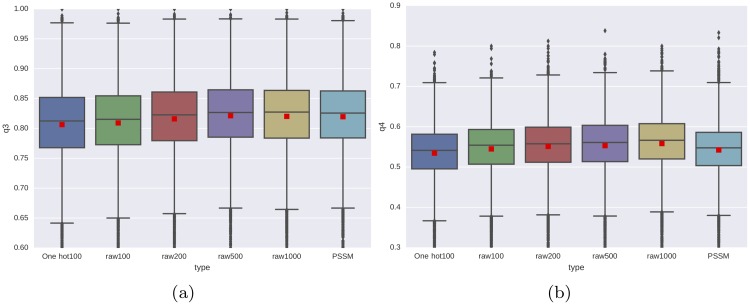
Per target secondary structure (a) and four-class solvent accessibility (b) accuracy for predictions using one hot encoding, a number of rawMSA networks, and a classical PSSM network trained and tested on the same dataset. One hot100 skips the rawMSA embedding step and encodes the alignments using one hot encoding, limited to the top100 alignments for memory reasons. Four different rawMSA networks are tested at variable MSA depths, using top 100, 200, 500 or 1000 alignments from the MSAs as input to the SS-RSA network. The average accuracies are shown as red squares.

We have used the five-fold cross-validation results to determine the testing accuracy for SS-RSA. To compare the rawMSA approach against a classic profile-based method, we trained a separate network by removing the bottom layers from the SS-RSA network (embedding and first 2D convolution/Pooling layer) and we trained it by using the PSSMs calculated from the HHblits alignments as inputs (PSSM network). In order to assess the usefulness of amino acid embeddings, we also train a rawMSA neural network without embedding layer, where the input alignments are transformed using a simple one-hot encoding of each amino acid (One-hot100 network). In this case, because of limits imposed by the amount of memory available on our testing machine, we had to limit the one-hot network to 100 alignments only. We tested and trained the PSSM and one-hot network in the same way we trained the other networks, both for SS and RSA. In Fig 3 we compare the performance of the PSSM and one-hot network against several rawMSA networks trained on different numbers of input sequences (100 to 1000 MSA sequences). The boxplot shows how the rawMSA networks with more input sequences perform generally better, with the rawMSA500 and rawMSA1000 networks performing slightly better than the classic PSSM network in predicting secondary structure, and all the rawMSA networks outperforming the PSSM network in predicting solvent accessibility. We also show that the rawMSA100 network outperforms the One-hot100 network both in the SS and RSA experiments.

The final SS-RSA network is an ensemble of six networks trained in five-fold cross-validation on 100 to 3000 input MSA sequences per target.

The results are shown in Table 1.

**Table 1:** Results for the SS-RSA networks trained to predict secondary structure and solvent accessibility.

Predictor		Accuracy
rawMSA SS ensemble		83.4
One-hot100 SS		80.5
rawMSA100 SS		80.7
rawMSA1000 SS		81.8
rawMSA1000 SS BLOSUM62 sort		80.3
rawMSA1000 SS shuffle		79.8
PSSM SS		81.7
rawMSA RSA ensemble	(4-class)	57.7
One-hot100 RSA	(4-class)	53.7
rawMSA100 RSA	(4-class)	55.0
rawMSA1000 RSA	(4-class)	56.1
PSSM RSA	(4-class)	54.1
rawMSA RSA ensemble	(2-class)	81.2

The number in the predictor name refers to the number of sequences from the MSA that were used; *BLOSUM62 sort* and *shuffle* refers to different sorting of the sequences in the MSA (see text for details).

It is difficult to make a direct comparison of rawMSA against other predictors in literature because of inevitable differences in the datasets. One example of this comes from secondary structure prediction systems, which have recently been reported to predict at accuracies (Q3) of up to 84% [66], yet we have not been able to find a recent study where the reported accuracy is supported by a proper splitting of the training and testing sets (see “Avoiding homolog contamination” paragraph). Running local versions of existing software does not solve that problem since it is not clear exactly which sequences were used for training. Also, in many cases a final network is trained using all available sequence, which means that any test is bound to be contaminated by homologous information. However, given the very large size of our test set, the rigorousness of our experimental setup, and the fact that rawMSA outperforms our own PSSM-based method, we believe that rawMSA compares favorably against the state of the art.

The convolutional layers of rawMSA depends on the order of the input sequences, since that will change the block of aligned positions from which features are extracted. To estimate the degree if this dependence we trained rawMSA with sequences sorted by sequence similarity using the BLOSUM62 similarity matrix (rawMSA1000 SS BLOSUM62 sort) and with randomly shuffled sequences (rawMSA1000 SS shuffle), see Table 1). Even though the performance decrease is small, neither of these two approaches worked as well as using the MSA as outputted by HHblits, most likely because it is easier to learn from blocks of similar positions with smoother mutational transitions.

### 3.3 CMAP predictions

The final CMAP network is an ensemble of 10 networks trained on 10 to 1000 input sequences and varying numbers of layers (10 to 24 convolutional layers). The CMAP predictions for each target have been sorted by the contact probability measure output by the ensemble, then the top *L*/5 long-range contacts have been evaluated against the native contact map. The final accuracy has been calculated as the average of the accuracies for all targets. In order to make a fair comparison against the other predictors, we have downloaded all of the predictions made in CASP12 and evaluated them with the same system. In Table 2 we compare the top *L*/5 long-range accuracy of rawMSA CMAP against the top 5 CASP12 predictors.

**Table 2:** Comparison of rawMSA against the top 5 contact prediction methods in CASP12.

Predictor	Domain Count	L/5 LR Accuracy
rawMSA CMAP	37	43.8
RaptorX-Contact	37	43.0
iFold_1	36	42.3
Deepfold-Contact	37	38.6
MetaPSICOV	37	38.4
MULTICOM-CLUSTER	37	37.9

All predictions from CASP12 have been re-evaluated to ensure a fair comparison. The accuracy is calculated on the top L/5 long-range contacts.

rawMSA outperforms the top predictors in CASP12 under the same testing conditions. This is unexpected, since it is the only top predictor not to use any kind of explicit coevolution-based features, or any other inputs than the MSA. On the other hand, CASP12 was held in 2016 and the field has made rapid progress since then. For example, the group behind RaptorX-Contact has reported an improvement of roughly 12 percentage points on this same CASP12 test set only months after the experiment was closed with a new deeper version of their neural network [44].

**Fig 4:**
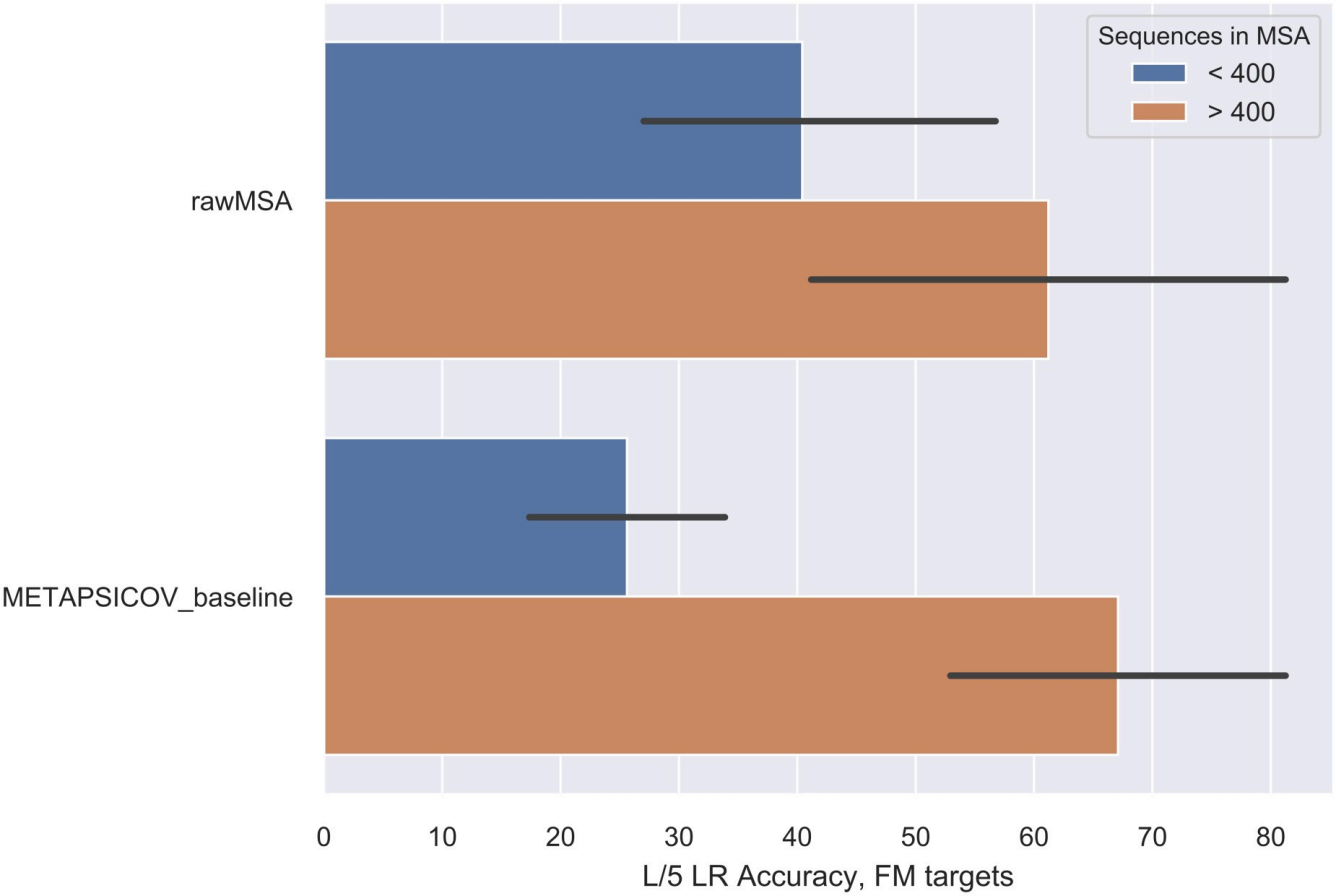
Impact of the number of sequences in the MSA on performance for rawMSA and METAPSICOV_baseline in the CASP13 FM targets.

Moreover, we expect rawMSA to be better than coevolution-based methods only whenever a relatively small number of sequences can be found in an MSA for a given target sequence, since only up to 1000 input MSA sequences could be used in training and testing because of limits in the amount of GPU RAM available (<25GB). Coevolution-based methods are more accurate as the number of sequences in the MSA increases and better metagenomic datasets [67] will produce larger MSAs for more target sequences. In the latest CASP13 experiment [68], where we participated with a prototype of rawMSA trained on a smaller ensemble of simpler models (up to 400 sequences per MSA) using a smaller sequence databases (Uniclust30), rawMSA was not among the best predictors in the contact prediction category. Nevertheless, the rawMSA prototype still outperformed a number of other coevolution-based methods, in particular the METAPSICOV_baseline method for MSAs with < 400 sequences, see Fig 4. For sequences with > 400 sequences in the MSA METAPSICOV_baseline method is still better. However, since we observe a clear correlation in testing accuracy and the number of sequences used as input, it is reasonable to expect that rawMSA will benefit from training on GPUs with larger memory allowing more sequences and deeper architectures to be used. In addition, rawMSA CMAP could also be improved by predicting distances, which seem to be direction in which the field is heading.

## 4 Conclusion

We have presented a new paradigm for the prediction of structural features of proteins called rawMSA, which involves using raw multiple sequence alignments (MSA) of proteins as input instead of compressing them into protein sequence profiles, as is common practice today. Furthermore, rawMSA does not need any other manually designed or otherwise hand-picked extra feature as input, but instead exploits the capability that deep networks have of automatically extracting any relevant feature from the raw data.

To convert MSAs, which could be described as categorical data, to a more machine-friendly format, rawMSA adopts embeddings, a technique from the field of Natural Language Processing to adaptively map discrete inputs from a dictionary of symbols into vectors in a continuous space.

To showcase our novel representation of the MSA, we developed a few different flavors of rawMSA to predict secondary structure, relative solvent accessibility and inter-residue contact maps. All these networks use the same and only kind of input, i.e. the MSA. After rigorous testing, we show how rawMSA SS-RSA sets a new state of the art for these kinds of predictions, and rawMSA CMAP performs on par with methods using more pre-calculated features in the inter-residue contact map prediction category in CASP12 and CASP13. Clearly demonstrating that rawMSA represents a promising development that can pave the way for improved methods using rawMSA instead of sequence profiles to represent evolutionary information in the coming years.
